# High severity of abortion complications in fragile and conflict-affected settings: a cross-sectional study in two referral hospitals in sub-Saharan Africa (AMoCo study)

**DOI:** 10.1186/s12884-023-05427-6

**Published:** 2023-03-04

**Authors:** Estelle Pasquier, Onikepe O. Owolabi, Tamara Fetters, Richard Norbert Ngbale, Mariette Claudia Adame Gbanzi, Timothy Williams, Huiwu Chen, Claire Fotheringham, Daphne Lagrou, Catrin Schulte-Hillen, Bill Powell, Elisabeth Baudin, Veronique Filippi, Lenka Benova

**Affiliations:** 1 Epicentre - Médecins Sans Frontières, 34, avenue Jean Jaurès, 75019 Paris, France; 2 Department of Public Health and Primary Care, Ghent University, Ghent, Belgium; 3 Department of Public Health - Institute of Tropical Medicine, Antwerp, Belgium; 4 Guttmacher Institute, New York, USA; 5 Vital Strategies, New York, USA; 6 Ipas, Chapel Hill, NC USA; 7 Ministère de la santé et de la Population de la République Centrafricaine, Bangui, Central African Republic; 8 Epicentre - Médecins Sans Frontières, Jigawa State, Nigeria; 9 Médecins Sans Frontières, Sydney, Australia; 10 Médecins Sans Frontières, Brussels, Belgium; 11 Médecins Sans Frontières International, Geneva, Switzerland; 12 Faculty of Epidemiology and Population Health - London School of Hygiene and Tropical Medicine, London, UK

**Keywords:** Maternal health, Abortion, Postabortion care, Hospital, Armed conflict, Humanitarian, Fragile, Nigeria, Central African Republic

## Abstract

**Background:**

Abortion-related complications are one of the five main causes of maternal mortality. However, research about abortion is very limited in fragile and conflict-affected settings. Our study aims to describe the magnitude and severity of abortion-related complications in two referral hospitals supported by Médecins Sans Frontières and located in such settings in northern Nigeria and Central African Republic (CAR).

**Methods:**

We used a methodology similar to the World Health Organization (WHO) near-miss approach adapted in the WHO multi-country study on abortion (WHO-MCS-A). We conducted a cross-sectional study in the two hospitals providing comprehensive emergency obstetric care. We used prospective medical records’ reviews of women presenting with abortion-related complications between November 2019 and July 2021. We used descriptive analysis and categorized complications into four mutually exclusive categories of increasing severity.

**Results:**

We analyzed data from 520 and 548 women respectively in Nigerian and CAR hospitals. Abortion complications represented 4.2% (Nigerian hospital) and 19.9% (CAR hospital) of all pregnancy-related admissions. The severity of abortion complications was high: 103 (19.8%) and 34 (6.2%) women were classified as having severe maternal outcomes (near-miss cases and deaths), 245 (47.1%) and 244 (44.5%) potentially life-threatening, 39 (7.5%) and 93 (17.0%) moderate, and 133 (25.6%) and 177 (32.3%) mild complications, respectively in Nigerian and CAR hospitals. Severe bleeding/hemorrhage was the main type of complication in both settings (71.9% in the Nigerian hospital, 57.8% in the CAR hospital), followed by infection (18.7% in the Nigerian hospital, 27.0% in the CAR hospital). Among the 146 women (Nigerian hospital) and 231 women (CAR hospital) who did not report severe bleeding or hemorrhage before or during admission, anemia was more frequent in the Nigerian hospital (66.7%) compared to the CAR hospital (37.6%).

**Conclusion:**

Our data suggests high severity of abortion-related complications in these two referral facilities of fragile and conflict-affected settings. Factors that could contribute to this high severity in these contexts include greater delays in accessing post-abortion care, decreased access to contraceptive and safe abortion care that result in increased unsafe abortions; as well as increased food insecurity leading to iron-deficiencies and chronic anaemia. The results highlight the need for better access to safe abortion care, contraception, and high quality postabortion care to prevent and manage complications of abortion in fragile and conflict-affected settings.

**Supplementary Information:**

The online version contains supplementary material available at 10.1186/s12884-023-05427-6.

## Background

Exposure to conflict and contextual fragility significantly increases the risk of maternal deaths [1]. Maternal mortality ratios in conflict settings are twice as high as in stable ones [2] and frequently reach levels above 1000 deaths/100,000 live births [3]. In such settings, the increase in maternal deaths has been attributed to the decrease in the availability of maternal and reproductive health services [4] as well as disruptions to the continuum of care [5] which leads to deaths from complications that could be easily treatable in stable settings [1]. Fragile and conflict-affected settings include a wide range of situations including contexts with institutional and social fragility, as well as contexts directly exposed to violent armed conflict. The World Bank defines fragile settings as contexts with particularly severe development challenges such as weak institutional capacity, poor governance or political instability, and where the risk of conflict, displacement and/or natural disasters is high [6]. The World Health Organization (WHO) describes fragile, conflict-affected and vulnerable settings as contexts experiencing a range of situations, including prolonged disruption of essential public services, humanitarian crises, protracted emergencies and/or armed conflicts [7].

Access to comprehensive abortion care, which consists of safe abortion care, post-abortion care and contraception, was identified as a priority intervention in the 2010 Inter-Agency Field Manual on Sexual and Reproductive Health (SRH) in Humanitarian Settings [8]. However, comprehensive abortion care is rarely on the agenda of government or non-governmental organizations responding to humanitarian crises. In particular, stakeholders do not consider abortion complications to be an important health problem in these contexts [9, 10]. Yet, abortion-related complications are one of the five main causes of maternal mortality worldwide [11] and most likely result from unsafe abortions — which are preventable [12]. In addition, women in fragile and conflict-affected settings are at higher risks of sexual violence and engagement in transactional sex. They are also more likely to experience disruption of contraceptive and safe abortion care services provision, exposing them to an increased risk of unwanted pregnancy and unsafe abortion [9, 13, 14]. This increase in exposure to unsafe abortion together with the lack of access to or delays in accessing post-abortion care may increase the magnitude and severity of abortion-related complications [15].

Evidence on abortion complications in stable limited-resource settings is increasingly available [16–18]. A WHO Multi-Country study on Abortion (WHO-MCS-A) in 11 African countries found that 9.4% of patients presenting for abortion complications in referral facilities (mainly in stable settings) had severe complications [18]. However, data and evidence about abortion, abortion-related complications, and abortion care in fragile and conflict-affected settings remain very limited [9]. This lack of evidence is due to several factors including the stigma associated with abortion, restrictive abortion laws in many of these contexts, the fear of security risks to researchers and/or providers of abortion services, and the challenges with collecting reliable data in such contexts. According to one recent systematic review [19], comprehensive abortion care indicators are the least reported indicators in monitoring and evaluation reports of SRH services in humanitarian settings (3% of all indicators). Available systematic reviews exploring access to [20], utilization of [21], or effectiveness of [22] SRH interventions or services in humanitarian settings found few studies on post-abortion care or safe abortion care [23–26]. None assessed the magnitude and severity of abortion-related complications in fragile and conflict-affected settings. This gap in evidence exacerbates its invisibility, limiting its recognition as a health issue to be addressed. As a result, evidence about abortion in fragile and conflict-affected settings was identified by researchers, practitioners [27–29] and WHO [30] as a priority research topic.

To inform abortion-related policies, guidelines, and interventions in fragile and conflict-affected settings, we conducted the *AMoCo* (Abortion-related Morbidity and mortality in fragile and Conflict settings) study in two such contexts. They were: Bangui in the Central African Republic (CAR); and Jigawa State in northern Nigeria. The objective of this paper is to describe the severity, magnitude, and types of abortion complications in two referral hospitals, one in each setting.

## Methods

### Study design

This article focuses on one component of the *AMoCo* study: a quantitative cross-sectional study conducted using a prospective medical record review among women with pregnancy losses before fetal viability. Additional file 1 describes all components of the mixed-method *AMoCo* study. This study is registered with ClinicalTrials.gov, NCT04331847.

For this study, we used a methodology similar to the WHO Multi-Country Study on Abortion (WHO-MCS-A) [31] to generate comparable results to their study conducted in 210 facilities with Comprehensive Emergency Obstetric Care (CEmOC) capacity in 11 African countries [18]. We collected additional data to be able to also generate estimates using sub-Saharan African context-appropriate severity criteria. Results are reported according to the STROBE guidelines [32].

### Study setting

We selected two referral hospitals supported by Medecins Sans Frontieres (MSF, a non-governmental organization) in two different types of fragile and conflict-affected settings: an urban setting in CAR (Bangui); and a rural setting in northern Nigeria (Jigawa State). Each setting had to meet the following criteria: hospitals were in areas where the security of participants and researchers could be guaranteed; hospitals had wide catchment areas (≥ 500,000 inhabitants); provided post-abortion care to at least 500 women per year; conducted > 1000 deliveries per year; and were capable of providing all CEmOC signal functions. Signal functions are key medical interventions needed to provide emergency obstetric care including: the capacity to remove retained products; provide blood transfusion; and conduct abdominal surgery [31]. MSF supported the provision of free comprehensive SRH care in both facilities.

CAR is a country of 5.4 million inhabitants, with 71% of the population living below the international poverty line in 2020 [33] and where 35% of women of reproductive age have not attended formal school [34]. In 2017, it had the fifth highest maternal mortality ratio in the world, with 829 maternal deaths per 100,000 live births [3]. Abortion complications were the primary cause of maternal deaths (25%) in a study conducted in six districts in 2010 [35]. A chronic civil war involving several non-state armed groups has ravaged CAR for decades, trapping the population in a cycle of indiscriminate violence; nearly 70% of the country remained under the control of armed groups in 2019 [36]. The country ranked sixth out of 178 states on the Fragile States Index in 2020 [37]. The study hospital, situated in an urban area of the capital Bangui, served a catchment population of approximately 505,000 people, including 160,000 internally displaced persons in 2017. The area of this hospital and its catchment population were regularly affected by armed attacks during the inclusion period of the participants in this study. In 2019, this hospital provided care to more than 10,400 women seeking childbirth care and almost 2500 women seeking post-abortion care [38]. In CAR, safe induced abortion is authorized by law before 8 weeks of pregnancy if the woman’s health is in danger, in case of fetal impairment, incest, rape, or when a minor is in a “serious distress state”, and if it is provided by a medical doctor [39].

Nigeria had an estimated population of 206 million inhabitants in 2020 [40] and had the fourth global highest maternal mortality ratio in 2017 at 917 per 100,000 live births [3]. The country ranked twelfth of 178 states on the Fragile States Index in 2021 [41]. The Nigerian study hospital is situated in Jigawa State, a poor rural state in northwest Nigeria where 87% of the population live below the poverty line [42]. In this State, the maternal mortality ratio is estimated to be 1012 per 100,000 live births [43] and 75% of women of reproductive age never attended formal school [44]. During the period of participants inclusion, the Jigawa State was in a fragile situation as defined by the World Bank [6]. Jigawa State has reported frequent floodings [45, 46], herders-farmers clashes, kidnappings, and influx of displaced people because of conflicts between different armed groups including Boko Haram, the Islamic State in West Africa Province and various communal militia in the neighboring States of Yobe, Katsina, and Borno [47]. The study hospital had a catchment population of about 665,000 inhabitants in 2020, but around 50% of patients came from outside the catchment area (including conflict-affected neighboring states like Yobe and Katsina) [48]. In 2019, it provided care to 9150 women seeking childbirth care and to around 500 women who sought post-abortion care [38]. In northern Nigeria, safe induced abortion is legal when the procedure aims to preserve the life of a pregnant woman, and when performed by qualified practitioners [39].

While both countries have recently developed national safe abortion care guidelines [49, 50], training for providers and access to safe abortion care remain very limited.

### Population

All women presenting to the study hospitals with any signs or symptoms of pregnancy-loss-related complications or deaths at discharge were eligible for inclusion. Pregnancy loss included spontaneous/induced abortion, ectopic pregnancy, or molar pregnancy before fetal viability (28 weeks of gestation). Women with threatened abortion (defined as vaginal bleeding with a closed cervix) were excluded. In accordance with the Council for International Organizations of Medical Sciences guidelines [51], an informed consent opt-out procedure was set up in each facility, and women who opted out were excluded. This study reports on the results of the medical record review among women with complications from either a spontaneous or induced abortion. Additional file 2 describes the Prospective Medical Record Review methodology in detail.

The sample size was computed to estimate with precision the proportion of severe maternal outcome, which includes near-miss cases and deaths, among all women presenting with abortion complications in each study hospital. The minimum target sample size was 430 women with abortion complications.

### Data collection and management

The included participants presented between February 2020 and July 2021 at the Nigerian hospital (with an interruption between April and July 2020 due to COVID-19) and between November 2019 and January 2020 at the CAR hospital.

Using a list of standard key words about symptoms and diagnoses (for example “vaginal bleeding”, “abortion”, “sepsis”), trained study clinicians screened the triage, gynecology/obstetric, and intensive care wards’ registers daily to identify potentially eligible women. They reviewed medical records of these women with a standardized eligibility form and included them in the study the same day as their presentation if they were eligible and did not opt out. Thereafter, they extracted data from their MSF standardized PAC medical records daily till their admission ended, consulting the clinician in charge of their medical management. They recorded sociodemographic data, reproductive history, obstetric characteristics, clinical signs and symptoms, laboratory markers, medical management, clinical outcomes, and status at discharge. We also collected aggregate data on the weekly number of live births and pregnancy-related admissions from each hospital’s health information system to calculate the magnitude endpoints. Both hospitals use the same MSF maternal health information system including standardized PAC medical records, variable definitions, and monitoring systems.

Quality assurance procedures were implemented to ensure the collection of high-quality data. In summary, all standardized data collection tools, and procedures were piloted and revised. Standardized detailed definitions of each severity criteria and types of complications were used to collect consistent and comparable data in both study sites (see definitions in Table 1, Additional file 3 and Table 4). The full details of our quality assurance process can be found in Additional file 2. The study staff received an initial 2-weeks training and refresher trainings when participant inclusion was stopped and restarted due to COVID-19. Tracking of eligible women was done twice a day by the two different study clinicians to ensure that no eligible women were missed. They also checked all collected data against the corresponding medical record on site twice. The physicians on the central study team (CF, EP, HC, OO) performed additional monitoring and supervision remotely and on site especially on the data needed to diagnose types of complications and assign severity classifications to ensure comparability across study sites. Any identified data inconsistencies were corrected.

**Table 1:** WHO-MCS-A severity classification of abortion complications [18] and adaptations to reflect the Sub-Saharan Africa healthcare context

Mutually exclusive	WHO-MCS-A severity classification^a^	Adaptations made in our study^a^
**Mild complications**	• abnormal physical examination findings on initial assessment (vital signs, appearance, mental status, abdominal examination, gynaecological examination)	Same
**Moderate complications**	• severe bleeding, • abdominal syndrome, • and/or uterine infection	Same
**Potentially Life-Threatening Complications (PLTC):**	• severe systemic infection, • uterine perforation • and/or severe haemorrhage	Same with these adaptations: • severe haemorrhage: o adding a threshold for systolic blood pressure < 100 mmHg o adding bleeding + Hb < 4 g/dL to the definition • adding the following conditions: o generalized peritonitis o other intra-abdominal perforation
**Severe Maternal Outcome (SMO)**	• deaths at discharge • + near- miss cases defined by the WHO near-miss criteria [52] for organ dysfunction of either one or more of the following: cardiovascular, respiratory, renal, hepatic, neurological, uterine or coagulation/hematologic dysfunction (with transfusion of ≥5 units of blood)	Same except coagulation/hematologic dysfunction using transfusion of ≥2 units of blood as a threshold

^a^ Detailed definitions of each condition in both original and adapted WHO-MCS-A classifications are in Additional file 3

### Data analysis

The magnitude of abortion-related complications was estimated using two indicators: the number of abortion-related admissions per 100 pregnancy-related admissions and per 1000 live births. Pregnancy- or abortion-related admissions were defined as all women who presented to the hospital for a pregnancy- or abortion-related reason respectively and for whom a medical record was opened.

Based on clinical, laboratory and management-based indicators identified at presentation or during hospitalization, the severity of abortion-related complications was classified into four mutually exclusive categories of progressively higher severity in line with the WHO-MCS-A classification [18]: mild complications, moderate complications, potentially life-threatening complications (PLTCs), and severe maternal outcomes (SMO). Women were classified into the highest level of severity for which they met the criteria. SMOs include near-miss cases and deaths (based on woman’s status at discharge). An abortion-related near-miss case is a woman who nearly died but survived a life-threatening complication that occurred during a spontaneous or induced abortion or within 42 days of the end of the pregnancy [52, 53]. It was defined using the 25 WHO near-miss criteria [52]. PLTCs and SMOs constituted “severe complications”.

For the primary estimates generated from our analysis, some adaptations were made to the WHO-MCS-A classifications to calculate estimates reflecting the Sub-Saharan Africa healthcare context. The WHO-MCS-A classification with these adaptations is called the “Sub-Saharan Africa (SSA) adapted WHO-MCS-A classification” for the rest of the paper. To classify a woman in the SMO category, we used a cut-off of two or more units of blood transfused instead of five for the hematologic/coagulation dysfunction criteria as recommended by Tura et al. [54] and other African abortion studies [53, 55]. To classify a woman in the PLTC category, “any bleeding and haemoglobin <4g/dL”, “generalized peritonitis” and “other intra-abdominal perforations” were added to the criteria of PLTC as recommended by other abortion studies [53, 55, 56]. In addition, we noted that no systolic blood pressure (SBP) threshold was indicated in WHO-MCS-A to classify a woman as having severe haemorrhage in the PLTC category. Therefore, we defined hypotension as a SBP < 100 mmHg as per Green et al. [57]. Table 1 summarizes the definitions of each of the four severity categories of the original WHO-MCS-A [18] and the adaptations made in this study.

We calculated the facility-based abortion-related mortality ratio, near-miss ratio, and mortality index for each facility as defined in the WHO near-miss approach guidelines [58]. Sensitivity analysis was conducted to compare our frequency distributions across the four severity categories, the facility-based near-miss ratio and mortality index with the WHO-MCS-A’s results using the original WHO-MCS-A criteria [18].

For each type/underlying condition of abortion complication reported (hemorrhage, infection, traumatism/perforation, anemia), the near-miss risk and the case fatality risk (CFR) were computed. The near-miss risk is the number of near-miss cases per 100 cases of each abortion complication type/underlying condition. And the CFR is the number of deaths per 100 cases of each complication type/underlying condition.

Gestational age at presentation was categorized as first trimester (fewer than 13 weeks), second trimester (13 weeks or more) and was estimated from weeks of gestation using the ultra-sound assessment as the reference assessment method. For those missing this information, we used the last menstrual period date or, if missing, the uterine size assessed by the provider, or if missing, the provider’s estimation of gestational age. Marital status was categorized as currently married or in union (married/living with a partner) or not (single/separated/divorced/widowed).

We performed descriptive analysis using Stata 16.0 software (College Station, Texas, USA). Sociodemographic, reproductive, obstetrics characteristics of the sample as well as the percentage of women in each severity category, the mortality, near-miss and magnitude indicators were described using summary statistics. We calculated 95% confidence interval (95% CI) using exact methods.

### Patient and public involvement

In each study hospital, a local steering committee involving members from the Ministries of Health and Social Affairs, local researchers, local administrative and religious leaders as well as women’s and civil society organizations participated in the study conduct oversee, interpretation of the results, and dissemination of the findings.

## Results

### Description of samples

**Fig. 1:**
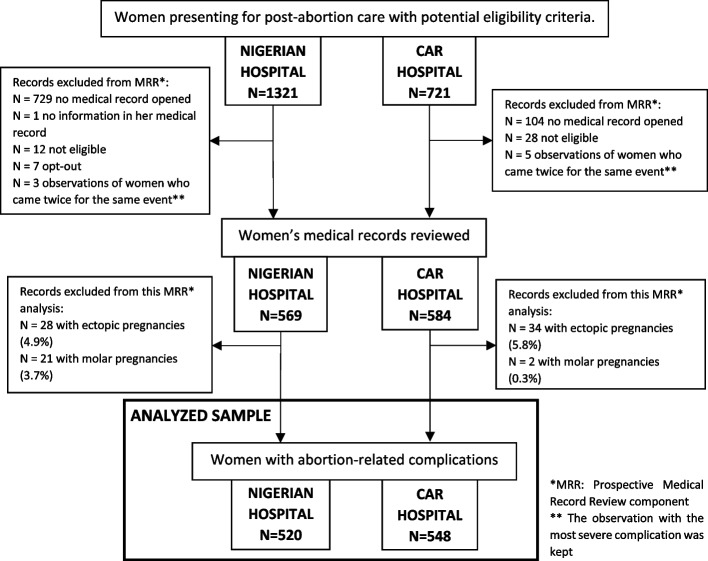
Study flow charts for abortion complications in the Nigerian and CAR study hospitals

Figure 1 describes the study flow charts of both study hospitals. In the Nigerian hospital, among the 1321 potentially eligible women, 520 women with abortion complications were included. In the CAR hospital, among the 721 potentially eligible, 548 women with abortion complications were included.

### Sociodemographic, obstetrics and reproductive characteristics

Table 2 shows the sociodemographic, admission, reproductive and obstetric characteristics of included women. Sociodemographic characteristics differed across the two study hospitals. While most women in both hospitals were between 20 and 29 years old (43.3% in the Nigerian hospital and 51.5% in the CAR hospital), a lower percentage of women in the Nigerian hospital were < 19 years (16.7%) compared to the CAR hospital (26.0%). In the Nigerian hospital, the majority (81.9%) were married, while in the CAR hospital, most women were unmarried (69.7%). Similar percentages of patients were referred from other facilities to both hospitals (15.5% in the Nigerian hospital and 12.6% in the CAR hospital). The reproductive histories of women in the Nigerian and CAR hospitals were similar. Most women had at least one previous pregnancy (83.0% in the Nigerian hospital and 75.1% in the CAR hospital) and had never had a previous abortion (69.1% in the Nigerian hospital and 77.3% in the CAR hospital). Few had previous uterine surgeries (5.2% in the Nigerian hospital and 4.7% in the CAR hospital). In contrast, the gestational age of the index pregnancy was different; 61.5% of the women presented in their second trimester of pregnancy in the Nigerian hospital compared to 32.9% in the CAR one.

**Table 2:** Sociodemographic, reproductive and obstetrics characteristics of women with abortion complications in Nigeria and CAR hospitals

	Nigerian hospital (*N*=520)			CAR hospital (*N*=548)		
	n	%	95%CI	n	%	(95%CI)
**Socio-demographic and admissions characteristics**						
**Age group (years)**	***N*** **= 520**			***N*** **= 546** (missing: 2)		
≤ 19	87	16.7	13.6–20.2	142	26.0	22.4–29.9
20–29	225	43.3	39.0–47.7	281	51.5	47.2–55.7
≥ 30	208	40.0	35.8–44.4	123	22.5	19.1–26.3
**Marital status**	***N*** **= 293** (missing: 227)			***N*** **= 353** (missing: 195)		
Not currently married or in union	53	18.1	13.9–23.0	246	69.7	64.6–74.4
Currently married or in union	240	81.9	77.0–86.1	107	30.3	25.6–35.4
**Hospitalization (stayed at least overnight)**	***N*** **= 520**			***N*** **= 548**		
Yes	441	84.8	81.4–87.8	511	93.3	90.8–95.2
**Referred from another facility**	***N*** **= 381** (missing: 139)			***N*** **= 538** (missing: 10)		
Yes	59	15.5	12.0–19.5	68	12.6	10.0–15.7
**Reproductive history and obstetric characteristics**						
**Previous pregnancies**	***N*** **= 517** (missing: 3)			***N*** **= 547** (missing:1)		
0	88	17.0	13.9–20.5	136	24.9	21.3–28.7
1 or more	429	83.0	79.5–86.1	411	75.1	71.3–78.7
**Previous abortions**	***N*** **= 517** (missing: 3)			***N*** **= 547** (missing:1)		
0	357	69.1	64.9–73.0	423	77.3	73.6–80.8
1 or more	160	30.9	27.0–35.1	124	22.7	19.2–26.4
**Previous uterine surgeries**	***N*** **= 520**			***N*** **= 548**		
0	493	94.8	92.5–96.6	522	95.3	93.1–96.9
1 or more	27	5.2	3.5–7.5	26	4.7	3.1–6.9
**Gestational age (in weeks)**	***N*** **= 488** (missing: 32)			***N*** **= 514** (missing: 34)		
< 13	188	38.5	34.2–43.0	345	67.1	62.9–71.2
13–28	300	61.5	57.0–65.8	169	32.9	28.8–37.1

### Magnitude

Table 3 shows that in the CAR hospital, the magnitude of abortion complications constituted nearly 19.9% of all pregnancy-related admissions; it was lower in the Nigerian hospital (4.2%).

**Table 3:** Magnitude of abortion complications in Nigeria and CAR study hospitals during the period of inclusion in the study

	Nigerian hospital^a^		CAR hospital^b^	
Total number of abortion-related admissions	520		548	
Total number of facility pregnancy-related admissions	12,332		2750	
Total number of live births in facility	6903		2018	
	**Estimate**	**95%CI**	**Estimate**	**95%CI**
Number of abortion admissions (per 100 pregnancy-related admissions)	4.2	3.9–4.6	19.9	18.5–21.5
Number of abortion admissions (per 1000 live births)	75.3	69.2–81.8	271.6	252.2–291.5

^a^Period of inclusion: February 2020 – July 2021 (with an interruption between April and July 2020 due to COVID-19)

^b^Period of inclusion: November 2019–January 2020

### Severity

**Fig. 2:**
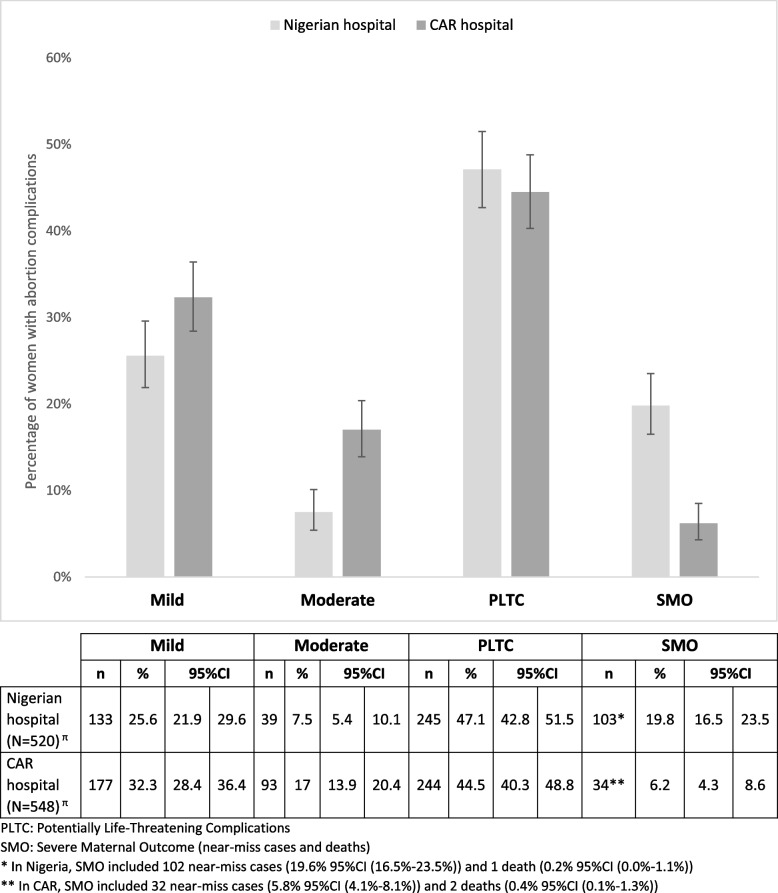
Proportion of women with abortion complications by severity category. (Sub-Saharan Africa adapted WHO-MCS-A classification)

Using the SSA adapted WHO-MCS-A classification, Fig. 2 shows high severity of abortion-related complications in both contexts with a higher proportion of severe maternal outcome in the Nigerian hospital compared to the CAR one. Two thirds of women in the Nigerian hospital (66.9%) and half in the CAR one (50.7%) had a severe complication including 19.8% of SMO in the Nigerian hospital versus 6.2% in the CAR one; and 47.1% PLTC in the Nigerian hospital versus 44.5% in the CAR one. The facility-based abortion-related near-miss ratio was 1478 per 100,000 live births in the Nigerian hospital and 1586 per 100,000 live births in the CAR one. The facility-based abortion mortality index was 1.0% in the Nigerian hospital and 5.9% in the CAR one. (Additional file 4).

Applying the original WHO-MCS-A classification, the sensitivity analysis showed that the percentage of women with severe complications remained unchanged but the distribution of the women in the SMO and PLTC categories differed. In particular, lower percentages of women were classified in the SMO category (4.6% in the Nigerian hospital and 3.8% in the CAR hospital) and higher percentages of women were in the PLTC category (62.3% in the Nigerian hospital and 46.9% in the CAR hospital) (Additional file 5). In this same sensitivity analysis, the facility-based abortion-related near-miss ratio was 333 per 100,000 live births in the Nigerian hospital and 942 per 100,000 live births in the CAR hospital. And the facility-based abortion mortality index was 4.2% in the Nigerian hospital and 9.5% in the CAR hospital. (Additional file 4).

### Types of complications

Table 4 shows that in both hospitals, the main type of complications was hemorrhage, with a higher proportion of women in the Nigerian hospital (71.9%) than in the CAR hospital (57.8%) experiencing it. Anemia and severe anemia were also more prevalent in the Nigerian hospital than in the CAR hospital (anemia: 82.0 and 42.7% respectively; severe anemia: 26.3 and 6.4% respectively). Notably, 66.7% of women in the Nigerian hospital and 37.6% of women in the CAR hospital had anaemia despite reporting no history of severe bleeding or haemorrhage before or during hospital admission.

**Table 4:** Main reported types of abortion complications and underlying conditions, their near-miss risk and CFR

(Not mutually exclusive)	Nigerian hospital				CAR hospital			
	Frequencies		Near-miss risk^f^	CFR^g^ (1 death)	Frequencies		Near-miss risk^f^	CFR^g^ (2 deaths)
	n/N (%)	(95%CI)	% (95%CI)	% (95%CI)	n/N (%)	(95%CI)	% (95%CI)	% (95%CI)
Severe bleeding/hemorrhage^a^	374/520 (71.9)	(67.8–75.8)	26.7 (22.3–31.5)	–	317/548 (57.8)	(53.6–62.0)	10.1 (7.0–14.0)	0.6 (0.1–2.3)
Infection^b^	97/520 (18.7)	(15.4–22.3)	44.3 (34.2–54.8)	1.0 (0.0–5.6)	148/548 (27.0)	(23.3–30.9)	14.2 (9.0–20.9)	1.3 (0.2–4.8)
Trauma/Perforation^c^	0/520 (0.0)	–	–	–	9/548 (1.6)	(0.8–3.1)	66.7 (29.9–92.5)	–
Anemia^e^ (Hb < 11 g/dL)	424/517 (82.0)^h^	(78.4–85.2)	23.6 (19.6–27.9)	0.2 (0.0–1.3)	219/513 (42.7) ^i^	(38.4–47.1)	14.2 (9.8–19.5)	0.9 (0.1–3.3)
*Anemia in women who had severe bleeding/hemorrhage*^d^	*328/373 (87.9)* ^h^	*(84.2–91.1)*	*29.9 (25.0–35.2)*	*–*	*140/303 (46.2)* ^i^	*(40.5–52.0)*	*22.1 (15.6–30.0)*	*1.4 (0.2–5.1)*
*Anemia in women who had no severe bleeding/hemorrhage*^d^	*96/144 (66.7)* ^h^	*(58.3–74.3)*	*2.1 (0.3–7.3)*	*1.0 (0.0–5.7)*	*79/210 (37.6)* ^i^	*(31.0–44.5)*	*7.6 (2.8–15.8)*	–
Severe anemia^e^ (Hb ≤ 7 g/dL)	136/517 (26.3) ^h^	(22.6–30.3)	52.2 (43.4–60.8)	–	33/513 (6.4) ^i^	(4.5–8.9)	66.7 (48.2–82.0)	6.0 (0.7–20.2)
*Severe anemia in women who had severe bleeding/hemorrhage*^d^	*134/373 (35.9)* ^h^	*(31.1–41.1)*	*52.2 (43.4–60.9)*	–	*33/303 (10.9)* ^i^	*(7.6–15.0)*	66.7 (48.2–82.0)	6.0 (0.7–20.2)
*Severe anemia in women who had no severe bleeding/hemorrhage*^d^	*2/144 (1.4)* ^h^	*(0.2–4.9)*	*50.0 (1.26–98.7)*	–	*0/210 (0)* ^i^	*–*	–	–

^a^Severe bleeding/hemorrhage includes severe vaginal bleeding (heavy bright red vaginal bleeding AND/OR pads/towels/clothing blood-soaked within 5 min AND/OR pallor with bleeding) and severe hemorrhage (blood loss > 1 L OR blood loss with  systolic blood pressure < 100 mmHg or requiring 1 unit of blood transfusion or with  hemoglobin < 4 g/dL)

^b^Infection includes uterine infections (fever/chills AND/OR foul-smelling vaginal discharge), generalized peritonitis (temperature > 38.5 °C AND abdominal guarding, rebound +/− ileus) and severe systemic infections (temperature > 38 °C AND suspected or confirmed infection AND at least one of the following: 1) new/worsened altered mentation. 2) respiratory rate ≥ 22/Min. 3) rystolic blood pressure ≤100 mmHg)

^c^Trauma/perforation includes evidence of cervix/vaginal mechanical injury at clinical examination, uterine perforation or other intra-abdominal perforation confirmed at laparotomy

^d^Severe bleeding/hemorrhage reported before presentation to the hospital and/or diagnosed during admission until discharge

^e^The threshold for anemia and severe anemia is defined according to WHO recommendations [59]

^f^The near-miss risk is the number of near-miss cases among the total number of cases of each type of abortion complications or underlying conditions

^g^*CFR: *the Case Fatality Risk is the number of deaths among the total number of cases of each type of abortion complications or underlying conditions

^h^Missing values < 2%

^i^ 4% < missing values < 10%

The reverse was the case for infection— a lower percentage of women had infection in the Nigerian hospital (18.7%) than in the CAR hospital (27.0%). In both sites, women with infection were more likely to experience a near-miss complication (44.3% in the Nigerian hospital and 14.2% in the CAR hospital) or die (CFR 1.3% in the Nigerian hospital and 1.0% in the CAR hospital) than women with hemorrhage (near-miss risk 26.7% in the Nigerian hospital and 10.1% in the CAR hospital and CFR 0.0% in the Nigerian hospital and 0.6% in the CAR hospital). In both sites, women with severe anemia had the highest near-miss risks (52.2% in the Nigerian hospital and 66.7% in the CAR hospital).

## Discussion

Our results suggest that women who accessed post-abortion services in two referral hospitals in fragile and conflict-affected settings experienced a higher frequency of severe abortion complications than women who used similar referral hospitals in more stable African settings. When applying the original WHO-MCS-A classification, the percentage experiencing severe complications (PLTC and SMO) in our study hospitals was 5.4 to 7.2 times that reported in the WHO-MCS-A African hospitals (9.4%) [18]. PLTC appeared to contribute a higher percentage of these severe complications than the SMOs. The facility-based near-miss ratio we estimated is also at least 2.5 times higher in our referral facilities than in 42 Nigerian tertiary hospitals in a recent study (137 per 100,000 live births) [60]. These latter results suggest that the population served by the study hospitals likely require more complex care to manage and survive their complications compared to stable settings.

Our results differ from results of a secondary analysis of the WHO-MCS-A data performed among 304 women of the Democratic Republic of Congo (DRC) showing no evidence that women in insecure areas were more likely to have a severe complication compared to women in secure areas (aOR 0.78; 95% CI, 0.34–1.79, *P* = 0.56) [26]. Authors of that study hypothesized that one possible reason was that unlike in secure areas, hospitals in insecure areas were mainly faith-based, where women may have been reluctant to seek postabortion care. Other published studies in DRC, Nigeria, and Burundi have found similar evidence, wherein fragile areas not directly affected by conflict may have maternal health indicators that are similar to or worse than those in conflict-affected areas in the same country. Their authors concluded that this phenomenon was likely due to minimal international support and investments in fragile areas [4, 47, 61–65] compared with the settings experiencing acute conflict.

We have several hypotheses that can be raised to explain the high severity in our study.

Evidence suggests that maternal health indicators deteriorate in fragile and conflict-affected areas as well as in their surrounding regions [2, 4, 47, 64] due to the disruption of health services. In addition, comprehensive abortion services are generally neglected within the SRH services provided in these contexts [9, 21, 22]. Furthermore, our two contexts regularly experience floods, especially Jigawa State [45, 46]. As documented elsewhere [66], flooding can also be a barrier to post-abortion care access by disrupting transportation thus limiting the availability of commodities. Therefore, health facilities within such settings are likely to have very limited post-abortion care capacity. As a result, women may seek care at multiple lower-level facilities that are unable to manage their post-abortion conditions, delaying their access to adequate post-abortion care. Similar to evidence from other studies [55, 67], these kinds of delays may have been more likely to occur in our Nigerian rural context than in our CAR urban setting due to more intense floodings, longer distances to reach hospitals, and additional challenges with obtaining transportation. On the other hand, one could also hypothesize that the armed attacks and the higher insecurity of the Bangui’s area prevented women’s displacement which in turn delayed their access to care. Nevertheless, some published studies suggest that the indirect effect of conflict, including the reduction of infrastructures and transportation, and the displacement of health staff contributes more to reduced access to maternal care than the direct conflict-related events [4, 64, 68].

Although Bangui appears to be more directly affected by conflict and insecurity than Jigawa, the severity of abortion complications is higher in the Jigawa hospital than the Bangui one. We believe that in this case, the combination of being a rural, poorer community [42] with exposure to more frequent natural disasters and receipt of lower support from international stakeholders [47], might have substantially increased the delays in access to care for women around the Nigerian hospital and thus the overall severity of abortion complications.

Expanding the provision of quality post-abortion care in these settings would reduce delays in access to care and prevent severe complications and deaths. Primary health centers should be enabled to provide post-abortion care using misoprostol, manual vacuum aspiration, and adequate antibiotic therapy as has been successfully implemented in other conflict-affected settings [69]. In addition, a strong referral system including free transportation between health facilities would increase accessibility.

Multiple studies suggest that contexts with restrictive abortion laws and policies [17, 56, 70, 71]—similar to ours [39]—are more likely to report a high burden of severe abortion complications*.* Research evidence shows that in these settings, several factors increase the risk of using unsafe methods to induce abortion and further lead women to delay seeking care for complications when they occur [72]. In particular, the stigma associated with providing and obtaining abortions is greater, providers have poor knowledge of the abortion law and are less likely to provide abortion care, and women lack access to accurate information on safe abortion care. The high severity of the complications in both study hospitals suggests that a significant number of women with severe complications may have undergone unsafe abortions. This is likely because access to SRH services including contraception and safe abortion care is very limited in fragile and conflict-affected contexts [73, 74]. The low prevalence of modern contraception among women of reproductive age in Bangui (33% [34]) and in Jigawa state (4% [44]) with unmet needs of contraception at 31% in Bangui [34] and 15% in Jigawa State [44], support this hypothesis. Furthermore, the proportion of infections was higher in our study than in the WHO-MCS-A study, especially in the CAR hospital (27.0% (95%CI: 23.3–30.9) versus 18.7% (95%CI:15.4–22.3) in the Nigerian hospital and 7.1% (95%CI: 6.7–7.6) in the African WHO-MCS-A study [75]). This high proportion of infection, especially in the Bangui’s hospital, suggests that many women may be using unsafe instrumental induced abortion [76].

Our findings suggest that increasing access to services that provide contraception and safe abortion care would decrease severe abortion morbidity in these contexts. For care to be successful, we recommend that health providers are trained to provide non-judgemental and respectful care, and that contraception and safe abortion care is decentralized to primary level facilities to increase access. Contraceptive services should provide both short- and long-acting methods, and safe abortion services should offer at least the recommended medication abortion methods. Emphasis should also be placed on improving women's and communities' access to quality information on safe and effective contraceptive methods and on how to use medication abortion safely. These efforts may also consider facilitating access to self-injectable contraceptives and medication abortion in the community as a harm reduction approach to avoid unsafe abortions [77].

Our results also highlight the possible critical impact of underlying chronic anemia on the prognosis of abortion-related complications in fragile and conflict-affected settings. The increased food insecurity leading to increased iron-deficiencies [4] in these contexts can be one factor that enhances chronic anemia which in turn, may aggravate complications of spontaneous and induced abortion in women with these co-existing conditions. 67% of Nigerian and 38% of CAR women included in our study and who did not have severe bleeding or hemorrhage reported before or during hospital admission, had anemia. These results suggest a high level of underlying chronic anemia in our populations, especially in Jigawa. Nigerian participants were more likely to experience severe hemorrhage than CAR participants (72% versus 58% respectively), which could be favored by this high level of underlying chronic anemia. Recent estimates from national health surveys support these hypotheses as they show that 46.8% of women of reproductive age (WRA) have anemia in CAR [78] and 65.4% of WRA in Jigawa State [44]. Those latter have some of the worst nutritional status indicators in the country: the second highest proportion of women with a height below 145 cm; the second highest proportion of women with a Body Mass Index below 18.5; and the highest proportion of women of reproductive age with severe anemia (4.2%) [44].

Although some studies suggest that there is an association between women’s sociodemographic characteristics and abortion-complications severity [18, 55, 79], the distributions of women’s age and marital status in our study do not seem to explain the higher severity in the Nigerian hospital. We do however, document that more women in the Nigerian hospital were admitted in second trimester of pregnancy compared to the CAR hospital. The literature shows that abortion-related complications in second trimester are associated with greater severity of complications [18, 55, 79]. This may also explain the higher severity of complications we report in the Nigerian site.

Interestingly, the magnitude of abortion complications is much higher in the CAR hospital (20% of all pregnancy-related admissions) compared to the Nigerian one (4.2%). Even though the health information systems in both hospitals use the same standardized MSF records, this difference between these estimates could also be linked to differences in the comprehensiveness of pregnancy-related admissions recording in each hospital. Nevertheless, our results are similar to previous studies, finding that abortion complications represented 4.4% of pregnancy-related admissions in 42 Nigerian tertiary hospitals [60] and 13.6% in another Bangui hospital [80]. Additional studies on maternal deaths in these countries have estimated that abortion complications were the primary cause of maternal mortality in CAR, accounting for almost 25% of all maternal deaths in 2010 [35], and the fifth leading cause in Nigeria, accounting for 8% of maternal deaths in 2016 [81].

Our results suggest that both study settings provide good quality of care for abortion-related complications as evidenced by the mortality index in both hospitals (9.5% in the CAR hospital and 4.2% in the Nigerian hospital), which are substantially lower than the 18.3% in the facilities in the WHO-MCS-A [18] and 34% in the nationwide Nigerian study [60]. The long-term MSF support to the two study hospitals which includes: health workers training; infrastructure strengthening; and the provision of free care can explain these good outcomes.

### Strengths and limitations

Despite the challenges in conducting abortion-related research in fragile and conflict-affected contexts, our study collected high quality data on the severity and magnitude of abortion complications of women presenting to referral hospitals in fragile and conflict-affected settings. Maternal Health research studies in low-resource environments have shown that data from facility records reviews can be unreliable, incomplete, or inaccurate especially when they are extracted retrospectively [16]. Although we cannot completely guarantee that the data collected at both hospitals were error-free, we believe that the use of standardized PAC medical records and the thorough application of the WHO-MCS-A standardized prospective methodology allows for comparison of the estimates of severity and types of complications from both hospitals. In particular, the identification and prospective follow up of women to determine complications severity and types of complications, the detailed clinical data collected prospectively with the trained clinicians in charge of the women using standardized detailed definitions and the inclusion of rigorous monitoring processes improved the reliability, validity, and comparability of our findings. The strong quality control procedures allowed us to minimize misclassifications of severity and types of complications, and missing values were minimal for both estimates. In addition, we collected all necessary data to be able to apply the SSA adapted WHO-MCS-A classification to present meaningful results for African context as well as the original WHO-MCS-A classification to allow some comparison with their results.

That said, our study has several important limitations. Because the insecure contexts obliged us to collect data in only one referral hospital in each setting, our results can’t be generalized to all referral facilities in Bangui, Jigawa State, Nigeria, CAR, or other fragile and conflict-affected settings. This also means that our results are not completely comparable with the WHO-MCS-A results which collected data in 10 referral facilities per randomly sampled geographical area. The lower proportion of women in the moderate category compared to the PLTC category reported in our study, is atypical and difficult to explain. We hypothesize that this might be due to differences in some detailed definitions in our study compared to the WHO study, particularly between the SBP thresholds used by clinicians for defining hypotension in the PLTC category. Our study introduced a threshold to define hypotension (SBP < 100 mmHg), whereas the WHO study definition did not. Clinicians in the WHO study facilities may have used varying SBP thresholds lower than the 100 mmHg in our study. There has been less research done to generate standardized and validated definitions with clear benchmarks for clinical signs and symptoms in the PLTC and moderate severity categories. Lastly, our results are not representative of the underlying population. While it can be hypothesized that near-miss abortion cases are only found in hospitals because they could not survive in the community without hospital care, all women who died in the community or who did not seek care or had complications managed in lower-level health facilities are not included in our samples.

Despite these limitations, we believe that our study highlights an important but neglected health challenge faced by women in such settings and showed it is feasible to conduct rigorous research on abortion in this context.

## Conclusion

Our data showed a high severity of abortion-related complications in these two referral hospitals in fragile and conflict-affected settings. Factors that may contribute to this high severity include disruption and dislocation of the health system; delays in accessing post-abortion care, non-prioritization of post-abortion care, safe abortion care and contraception as key sexual and reproductive health services; increased unsafe abortions; and increased food insecurity leading to iron-deficiencies and chronic anaemia. Our results highlight the need for greater access to contraception, safe abortion care, and high quality postabortion care to prevent and manage complications of abortion in these fragile and conflict-affected settings. In addition, preventing and managing underlying chronic health conditions like malnutrition and chronic anemia may reduce the lethality of abortion complications. Settings which are perhaps less directly exposed to conflicts, but which are in a less visible but significant state of chronic fragility or protracted emergency should not be forgotten. Humanitarian stakeholders and researchers should further quantify the burden of abortion complications in these settings and identify the specific barriers that prevent women and girls from accessing comprehensive abortion care to develop targeted actions that address their needs.

## Supplementary Information

**Additional file 1.** Full AMoCo study design.

**Additional file 2.** Additional information on the methodology of the prospective medical record review.

**Additional file 3.** WHO-MCS-A and adapted WHO-MCS-A severity classification.

**Additional file 4.** Facility-based near-miss ratio, mortality ratio and mortality index.

**Additional file 5.** Sensitivity analyses applying the original WHO-MCS-A severity classification.

## Data Availability

“The dataset collected during the study, including deidentified participant data, data dictionary and additional related documents such as study protocol, data collection tools, procedures and statistical analysis plan, are available from the corresponding author or dpco@epicentre.msf.org on reasonable request, following MSF’s data sharing policy which ensures that data will be available upon request to interested researchers while addressing all security, legal, and ethical concerns, especially for sensitive subjects like abortion in vulnerable populations (https://www.msf.org/sites/msf.org/files/msf_data_sharing_policycontact_infoannexes_final.pdf).”

## Abbreviations

*AMoCo*: Abortion-related morbidity and mortality in fragile and conflict-affected settings (study)
CAR: Central african republic
CEmOC: Comprehensive emergency maternal obstetric care
CFR: Case fatality risk
MRR: Prospective medical record review
PLTC: Potentially life-threatening complications
SMO: Severe maternal outcome
SRH: Sexual and reproductive health
SSA: Sub-saharan africa
WHO: World health organization
WHO-MCS-A: World health organization multi-country study on abortion
WRA: Women of reproductive age

## References

[1] Kotsadam A, Østby G. Armed conflict and maternal mortality: a micro-level analysis of sub-Saharan Africa, 1989–2013. Soc Sci Med. 2019;239:112526. 10.1016/j.socscimed.2019.112526 31520880

[2] Jawad M, Hone T, Vamos EP, Cetorelli V, Millett C (2021). Implications of armed conflict for maternal and child health: a regression analysis of data from 181 countries for 2000–2019. PLoS Med.

[3] WHO, UNICEF, UNFPA, World Bank Group, UNDP. Trends in maternal mortality 2000 to 2017. Geneva: World Health Organisation; 2019;122. https://apps.who.int/iris/bitstream/handle/10665/327596/WHO-RHR-19.23-eng.pdf?sequence=13&isAllowed=y. Accessed 08 Feb 2023.

[4] Garry S, Checchi F (2020). Armed conflict and public health: into the 21st century. J Public Health (Bangkok).

[5] Rammohan A, Mavisakalyan A, Vu L, Goli S (2021). Exposure to conflicts and the continuum of maternal healthcare: analyses of pooled cross-sectional data for 452,192 women across 49 countries and 82 surveys. PLoS Med.

[6] The World Bank (2019). Revised Classification of Fragility and Conflict Situations for World Bank Group Engagement.

[7] World Health Organization (2020). Quality of Care in Fragile , Conflict-Affected and Vulnerable Settings: Taking Action.

[8] Inter-agency Working Group on Reproductive Health in Crises (2010). Inter-agency Field Manual on Reproductive Health in Humanitarian Settings.

[9] Dias Amaral B, Sakellariou D (2021). Maternal health in crisis: a scoping review of barriers and facilitators to safe abortion Care in Humanitarian Crises. Front Glob Women’s Heal.

[10] McGinn T, Casey SE (2016). Why don’t humanitarian organizations provide safe abortion services?. Confl Health..

[11] Graham W, Woodd S, Byass P, Filippi V, Gon G, Virgo S (2016). Maternal health 1 - diversity and divergence: the dynamic burden of poor maternal health. Lancet..

[12] Grimes DA, Benson J, Singh S, Romero M, Ganatra B, Okonofua FE (2006). Unsafe abortion: the preventable pandemic. Lancet..

[13] Erhardt-Ohren L (2020). Refugee and internally displaced Women’s abortion knowledge, attitudes and practices: addressing the lack of research in low- and middle-income countries. Int Perspect Sex Reprod Health.

[14] UNFPA, UNHCR. Operational guidance – responding to the health and protection needs of people selling or exchanging sex in humanitarian settings. Geneva; 2021;84. https://www.unhcr.org/protection/operations/60dc85d74/responding-health-protection-needs-people-selling-exchanging-sex-humanitarian.html. Accessed 8 Feb 2023.

[15] Blanchet K, Roberts B (2015). An evidence review of research on health interventions in humanitarian crises.

[16] Adler AJ, Filippi V, Thomas SL, Ronsmans C (2012). Incidence of severe acute maternal morbidity associated with abortion: a systematic review. Trop Med Int Heal.

[17] Calvert C, Owolabi OO, Yeung F, Pittrof R, Ganatra B, Tunçalp Ö (2018). The magnitude and severity of abortion-related morbidity in settings with limited access to abortion services: a systematic review and meta-regression. BMJ Glob Heal..

[18] Qureshi Z, Mehrtash H, Kouanda S, Griffin S, Filippi V, Govule P (2021). Understanding abortion-related complications in health facilities: results from WHO multicountry survey on abortion (MCS-A) across 11 sub-Saharan African countries. BMJ Glob Heal..

[19] Broaddus-Shea ET, Kobeissi L, Ummer O, Say L (2019). A systematic review of monitoring and evaluation indicators for sexual and reproductive health in humanitarian settings. Confl Health..

[20] Jennings L, George AS, Jacobs T, Blanchet K, Singh NS. A forgotten group during humanitarian crises: a systematic review of sexual and reproductive health interventions for young people including adolescents in humanitarian settings: BioMed Central Ltd.; 2019. 10.1186/s13031-019-0240-y PMC6880589 31788022

[21] Singh NS, Aryasinghe S, Smith J, Khosla R, Say L, Blanchet K (2018). A long way to go: a systematic review to assess the utilisation of sexual and reproductive health services during humanitarian crises. BMJ Glob Heal..

[22] Singh NS, Smith J, Aryasinghe S, Khosla R, Say L, Blanchet K (2018). Evaluating the effectiveness of sexual and reproductive health services during humanitarian crises: a systematic review. PLoS One.

[23] Casey SE, Chynoweth SK, Cornier N, Gallagher MC, Wheeler EE. Progress and gaps in reproductive health services in three humanitarian settings: mixed-methods case studies. Confl Health. 2015;9 Suppl 1:S3. 10.1186/1752-1505-9-S1-S3 PMC4331815 25798189

[24] Tran N-T, Dawson A, Meyers J, Krause S, Hickling C, Inter-Agency Working Group (IAWG) on Reproductive Health in Crisis I-AWG (IAWG) on RH in, et al. Developing institutional capacity for reproductive health in humanitarian settings: Public Library of Science; 2015. 10.1371/journal.pone.0137412 PMC4558004 26331474

[25] Foster AM, Arnott G, Hobstetter M (2017). Community-based distribution of misoprostol for early abortion: evaluation of a program along the Thailand-Burma border. Contraception..

[26] Wolomby-Molondo J-J, Calvert C, Seguin R, Qureshi Z, Tunçalp Ö, Filippi V (2021). The relationship between insecurity and the quality of hospital care provided to women with abortion-related complications in the Democratic Republic of Congo: a cross-sectional analysis. Int J Gynecol Obstet.

[27] Scott RH, Filippi V, Moore AM, Acharya R, Bankole A, Calvert C (2018). Setting the research agenda for induced abortion in Africa and Asia. Int J Gynecol Obstet.

[28] Singh NS, Ataullahjan A, Ndiaye K, Das JK, Wise PH, Altare C (2021). Delivering health interventions to women, children, and adolescents in conflict settings: what have we learned from ten country case studies?. Lancet..

[29] Kobeissi L, Nair M, Evers ES, Han MD, Aboubaker S, Say L (2021). Setting research priorities for sexual, reproductive, maternal, newborn, child and adolescent health in humanitarian settings. Confl Health..

[30] Kouanda S, Qureshi Z (2022). Quality of care for abortion-related complications: insights from the WHO multi-country survey on abortion-related morbidity across 11 African countries. Int J Gynecol Obstet.

[31] Kim CR, Tunçalp Ö, Ganatra B, Gülmezoglu AM (2016). WHO multi-country survey on abortion-related morbidity and mortality in health facilities: study protocol. BMJ Glob Heal..

[32] Vandenbroucke JP, von Elm E, Altman DG, Gøtzsche PC, Mulrow CD, Pocock SJ (2014). Strengthening the reporting of observational studies in epidemiology (STROBE): explanation and elaboration. Int J Surg.

[33] The World Bank. Central African Republic overview: development news, research, data | World Bank. World Bank 2021. https://www.worldbank.org/en/country/centralafricanrepublic/overview#1. Accessed 2 Mar 2022.

[34] ICASEES - RCA (2019). MICS6-RCA Enquête par grappes à indicateurs multiples 2018–2019, Rapport final des résultats de l’enquête.

[35] Ministere de la sante, UNFPA. Evaluation de la disponibilité, de l’utilisation et de la qualité des soins obstétricaux d’urgence dans la zone d’intervention de l’UNFPA en République Centrafricaine. Bangui; 2010.

[36] Human Rights Watch (2020). World Report 2020: Central African Republic | Human Rights Watch.

[37] Fund for Peace. Fragile States Index Annual Report 2020. Geneva; 2020. www.fundforpeace.org. Accessed 13 Feb 2023.

[38] MSF - OCB (2019). Activity data from MSF monitoring system (DHIS-II).

[39] WHO - Human Reproduction Programme - Research for impact. Countries Archive - Global Abortion Policies Database. 2018. https://abortion-policies.srhr.org/. Accessed 12 Feb 2023.

[40] The World Bank. Nigeria overview: development news, research, data | world bank. World Bank. 2021. https://www.worldbank.org/en/country/nigeria/overview#1. Accessed 3 Mar 2022.

[41] Fund for Peace. Fragile States Index Annual Report 2021. Geneva; 2021. www.fundforpeace.org. Accessed 13 Feb 2023.

[42] National Bureau of Statistics. 2019 Poverty and Inequality in Nigeria: Executive Summary. National Bureau of Statistics (NBS). 2020. pp. 1–27. https://nigerianstat.gov.ng/download/1092. Accessed 8 Feb 2023.

[43] Sharma V, Brown W, Kainuwa MA, Leight J, Nyqvist MB (2017). High maternal mortality in Jigawa state, northern Nigeria estimated using the sisterhood method. BMC Pregnancy Childbirth..

[44] National Population Commission (NPC) [Nigeria], ICF. Nigeria Demographic Health Survey 2018. 2019. p. 748. https://dhsprogram.com/publications/publication-fr359-dhs-final-reports.cfm. Accessed 8 Feb 2023.

[45] Yusuf Y, Ekpu G (2020). Nigeria: Jigawa state farmers lose “80% of farmland” due to flooding - BBC News BBC News.

[46] Jigawa State Government. Flooding - Jigawa state government. Government website 2017. https://www.jigawastate.gov.ng/flood.php. Accessed 24 Mar 2022.

[47] Tyndall JA, Ndiaye K, Weli C, Dejene E, Ume N, Inyang V (2020). The relationship between armed conflict and reproductive, maternal, newborn and child health and nutrition status and services in northeastern Nigeria: a mixed-methods case study. Confl Health..

[48] Médecins Sans Frontières. Project overview - Jigawa state: Médecins Sans Frontières; 2020.

[49] Ministère de la Santé et de la population - République Centrafricaine (2021). Directives nationales sur l’avortement sécurisé selon la loi et les soins après-avortement en République Centrafricaine.

[50] Federal Ministry of Health Nigeria. National-Guidelines-on-Safe-Termination-of-Pregnancy-for-Legal-Indications. Abuja; 2018;26. https://www.health.gov.ng/doc/National_Guidelines_on_Safe_Termination_of_Pregnancy.pdf. Accessed 12 Feb 2023.

[51] Council for International Organizations of Medical Sciences (CIOMS). International Ethical Guidelines for Health-related Research Involving Humans. 2016;119. https://cioms.ch/publications/product/international-ethical-guidelines-for-health-related-research-involving-humans/. Accessed 12 Feb 2023. 40523065

[52] World Health Organization. The WHO near-miss approach for maternal health. Geneva; 2011. p. 58. https://apps.who.int/iris/handle/10665/44692. Accessed 12 Feb 2023.

[53] Owolabi OO, Cresswell JA, Vwalika B, Osrin D, Filippi V (2017). Incidence of abortion-related near-miss complications in Zambia: cross-sectional study in central. Copperbelt Lusaka Provinces Contraception.

[54] Tura AK, Stekelenburg J, Scherjon SA, Zwart J, van den Akker T, van Roosmalen J (2017). Adaptation of the WHO maternal near miss tool for use in sub-Saharan Africa: an international Delphi study. BMC Pregnancy Childbirth.

[55] Madziyire MG, Polis CB, Riley T, Sully EA, Owolabi O, Chipato T (2018). Severity and management of postabortion complications among women in Zimbabwe, 2016: a cross-sectional study. BMJ Open.

[56] Bankole A, Kayembe P, Chae S, Owolabi O, Philbin J, Mabika C (2018). The severity and management of complications among postabortion patients treated in Kinshasa health facilities. Int Perspect Sex Reprod Health.

[57] Green LJ, Mackillop LH, Salvi D, Pullon R, Loerup L, Tarassenko L (2020). Gestation-specific vital sign reference ranges in pregnancy. Obstet Gynecol.

[58] World Health Organization, Organization WH (2011). Evaluating the quality of care for severe pregnancy complications: the WHO near-miss approach for maternal health.

[59] World Health Orgranization (WHO), WMNIS (2011). Haemoglobin concentrations for the diagnosis of anaemia and assessment of severity.

[60] Adanikin AI, Umeora OUJ, Nzeribe E, Agbata AT, Ezeama C, Ezugwu FO (2019). Maternal near-miss and death associated with abortive pregnancy outcome: a secondary analysis of the Nigeria near-miss and maternal death survey. BJOG An Int J Obstet Gynaecol..

[61] Malembaka EB, Altare C, Bigirinama RN, Bisimwa G, Banywesize R, Tabbal N (2021). The use of health facility data to assess the effects of armed conflicts on maternal and child health: experience from the Kivu, DR Congo. BMC Health Serv Res.

[62] Zhang T, Qi X, He Q, Hee J, Takesue R, Yan Y (2021). The effects of conflicts and self-reported insecurity on maternal healthcare utilisation and children health outcomes in the democratic republic of Congo (Drc). Healthc..

[63] Ziegler BR, Kansanga M, Sano Y, Kangmennaang J, Kpienbaareh D, Luginaah I (2021). Antenatal care and skilled birth in the fragile and conflict-affected situation of Burundi. Int J Health Plann Manag.

[64] Chukwuma A (2019). Ekhator-Mobayode UE.

[65] Altare C, Malembaka EB, Tosha M, Hook C, Ba H, Bikoro SM, et al. Health services for women, children and adolescents in conflict affected settings: experience from north and south Kivu, Democratic Republic of Congo. Confl Health. 2020;14:19. 10.1186/s13031-020-00265-1 PMC7254646 32514296

[66] Ray-Bennett NS, Corsel DMJ, Goswami N, Ghosh A. Understanding reproductive health challenges during a flood: insights from Belkuchi Upazila, Bangladesh. Gates Open Res. 2019;3:21. 10.12688/gatesopenres.12920.1 PMC6603260 31294418

[67] Kalilani-Phiri L, Gebreselassie H, Levandowski BA, Kuchingale E, Kachale F, Kangaude G (2015). The severity of abortion complications in Malawi. Int J Gynecol Obstet.

[68] Mayega RW, Tumuhamye N, Atuyambe L, Okello D, Bua G, Komakech D (2015). Qualitative assessment of resilience to the effects of chronic conflict in authors.

[69] Gallagher M, Morris C, Aldogani M, Eldred C, Shire AH, Monaghan E (2019). Postabortion care in humanitarian emergencies: improving treatment and reducing recurrence. Global Health Sci Pract.

[70] Ziraba AK, Izugbara C, Levandowski BA, Gebreselassie H, Mutua M, Mohamed SF (2015). Unsafe abortion in Kenya: a cross-sectional study of abortion complication severity and associated factors. BMC Pregnancy Childbirth..

[71] Srinil S (2011). Factors associated with severe complications in unsafe abortion. J Med Assoc Thail.

[72] Mehrtash H, Kim CR, Ganatra B, Tuncalp Ö (2021). What’s needed to improve safety and quality of abortion care: reflections from WHO/HRP multi-country study on abortion across the sub-Saharan Africa and Latin America and Caribbean regions. BMJ Glob Heal..

[73] McGinn T, Austin J, Anfinson K, Amsalu R, Casey SE, Fadulalmula S, et al. Family planning in conflict: results of cross-sectional baseline surveys in three African countries. Confl Health. 2011;5:8. 10.1186/1752-1505-5-11 PMC3162885 21752241

[74] Casey SE, Isa GP, Mazambi EI, Giuffrida MM, Kulkarni MJ, Perera SM. Community perceptions of the impact of war on unintended pregnancy and induced abortion in Protection of Civilian sites in Juba, South Sudan. 2022;17:2176–89. 10.1080/17441692.2021.1959939. Accessed 12 Feb 2023. 10.1080/17441692.2021.1959939 34323171

[75] Baguiya A, Mehrtash H, Bonet M, Adu-Bonsaffoh K, Compaoré R, Bello FA (2022). Abortion-related infections across 11 countries in sub-Saharan Africa: prevalence, severity, and management. Int J Gynecol Obstet.

[76] Sajadi-Ernazarova KR, Martinez CL. Abortion Complications. [Updated 2022 May 23]. StatPearls. 2022. https://www.ncbi.nlm.nih.gov/books/NBK430793/. Accessed 12 Feb 2023

[77] Jayaweera R, Powell B, Gerdts C, Kakesa J, Ouedraogo R, Ramazani U (2021). The potential of self-managed abortion to expand abortion access in humanitarian contexts. Front Glob Women’s Heal..

[78] The World Bank database. Prevalence of anemia among women of reproductive age (% of women ages 15–49) - Central African Republic, Nigeria | Data 2000–2019. 2019. https://data.worldbank.org/indicator/SH.ANM.ALLW.ZS?locations=CF-NG. Accessed 3 Mar 2022.

[79] Romero M, Ponce G, de Leon R, Baccaro L, Carroli B, Mehrtash H, Randolino J (2021). Abortion-related morbidity in six Latin American and Caribbean countries: findings of the WHO/HRP multi-country survey on abortion (MCS-A). BMJ Glob Heal.

[80] Sepou A, Ngbale R, Yanza MC, Domande-Modanga Z, Nguembi E (2004). Analyse des avortements a la maternité de l’hôpital communautaire de Bangui. Med Trop.

[81] Oladapo OT, Adetoro OO, Ekele BA, Chama C, Etuk SJ, Aboyeji AP (2016). When getting there is not enough: a nationwide cross-sectional study of 998 maternal deaths and 1451 near-misses in public tertiary hospitals in a low-income country. BJOG An Int J Obstet Gynaecol.

