# Supplementary material for: High severity of abortion complications in fragile and conflict-affected settings: a cross-sectional study in two referral hospitals in sub-Saharan Africa (AMoCo study)
Source: BMC Pregnancy Childbirth. 2023 Mar 4;23:143. doi: 10.1186/s12884-023-05427-6 (PMC9985077; doi:10.1186/s12884-023-05427-6)
Supplement: Supplementary file 1 — Additional file 1. Full AMoCo study design. [file 12884_2023_5427_MOESM1_ESM.pdf]

Additional file 1: Full AMoCo study design

The AMoCo[1] study included 4 components including:

- Among patients presenting for Post-Abortion Care:
    1) A quantitative cross-sectional observational study including
        - A prospective medical record review of all women presenting for abortion complications with a medical record.
        - A quantitative survey of these women who stayed at least overnight and survived
    2) A qualitative survey among the most severe patients (near-miss)
- In the health facility:
    1) A rapid facility assessment of its capability to provide comprehensive abortion care
    2) A Knowledge Attitudes Behavior and Practice survey about abortion care among health professionals involved in comprehensive abortion care

Figure Additional file 1: AMoCo Study Design

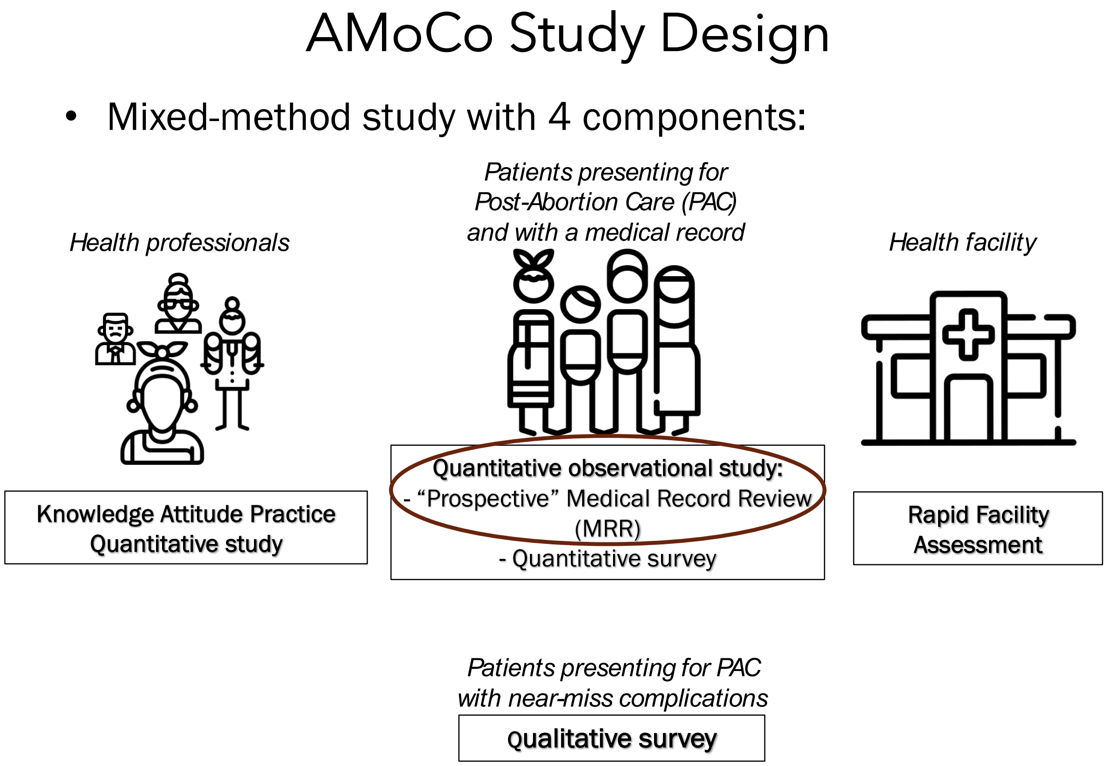

This first article focuses on the results of the prospective medical record review among women presenting with abortion complications. Future articles will provide the results of other components.

[1] **A**bortion-related **Mo**rbidity and mortality in fragile and **Co**nflict-affected setting study
