# Supplementary material for: High severity of abortion complications in fragile and conflict-affected settings: a cross-sectional study in two referral hospitals in sub-Saharan Africa (AMoCo study)
Source: BMC Pregnancy Childbirth. 2023 Mar 4;23:143. doi: 10.1186/s12884-023-05427-6 (PMC9985077; doi:10.1186/s12884-023-05427-6)
Supplement: Supplementary file 2 — Additional file 2. Additional information on the methodology of the prospective medical record review. [file 12884_2023_5427_MOESM2_ESM.pdf]

# Additional file 2: Additional information on the methodology of the prospective medical record review

*Sample sizes:*

The sample size of the Medical Record Review (MRR) was computed to estimate with precision the primary endpoint of the study: the proportion of near-miss cases and deaths (also described as severe maternal outcome) among all women presenting with abortion complications in each study site. Using the normal approximation to the binomial calculation(1) and based on our hypothesis that the proportion of SMO among these women will be approximately 12% or twice the expected proportion of 6% in stable contexts as published in available publications at the protocol writing stage(2,3), a sample of 350 women per site would allow an estimation with 3.5% precision and a type I error of 5% (95% confidence intervals). Estimating an attrition rate of 20%[1], we needed to include and review the medical record of 430 women presenting for post-abortion care (PAC) per site.

We also considered that the underlying hypothesis of an expected proportion of severe maternal outcome in stable contexts at 6% could be over or underestimated because the definitions of near-miss cases and denominators vary widely between publications. Therefore, we estimated the precisions of the proportion of severe maternal outcome if the latter would vary between 2% and 30% with this sample size. The precisions were still acceptable as they varied between 1.5% (for a proportion of SMO at 2%) to 4.8% (for a proportion of SMO at 30%).

The study was planned to be implemented in three countries including Nigeria, Central African Republic and Democratic Republic of Congo (North Kivu). The study design had to be adapted in the Democratic Republic of Congo study site because of increased insecurity (armed attacks) in addition to epidemic outbreaks (Ebola, covid-19) and natural disaster (volcano eruption) during the years for which we sought to begin data collection. In the DRC site, we were able to conduct the medical record review retrospectively (instead of prospectively), which resulted in sufficient and useful but incomparable data when compared to the Nigeria and CAR sites. The remaining components of the study could not be implemented without hiring study staff on site which was not possible in this Democratic Republic of Congo study site.

In the 2 study sites in which the full study could be implemented as planned (Nigeria and CAR), the sample size and/or saturation were not reached in the qualitative component when we reached 430 women included into the Medical Record Review. Therefore, in these two study sites, we increased the necessary sample size to 550 per site in the medical record review component allowing for improved quality and quantity of information in the qualitative component of both study sites.

*Procedures, data collection and management:*

*Study personnel:*

The AMoCo study team implementing the medical record review component included a central study team and two field study teams (one in each study site).

The central study team included the co-principal investigator, one co-investigator, one multisite study coordinator, 1 central person responsible for data management. The two investigators and the multisite study coordinator were medical doctors by background. One additional co-investigator, a senior gynecological obstetric specialist was involved to resolve clinical questions.

[1] Excluding ectopic and molar pregnancies from the main analysis as well as excluding women with abortion with unexploitable medical records.

Each field study team had a study coordinator, 2 study clinicians, and 2 data clerks to manage the MRR data collection. The CAR site had also a deputy study coordinator who supervised the study clinicians.

*Pre-test, pilot and training:*

For each study components, manual of procedures and Standard Operating Procedures (SOP) have been developed to ensure quality and standardizations of processes.

A particular care was taken to clearly define in detail the primary endpoints of the study: the severity of complications classifications (cf. Additional file 3) and the types of complications (table 4 of the main article) and to collect in a standardized way all clinical data needed to apply these definitions.
Standardized WHO-MCS-A definitions were used to be able to calculate severity estimates as comparable as possible to their estimates in the African referral hospitals of stable contexts (cf. Additional file 3). We noted that no systolic blood pressure (SBP) threshold was indicated in WHO-MCS-A to classify a woman as having severe haemorrhage in the PLTC category. Therefore, we defined hypotension as a SBP < 100 mm Hg as per Green et al[58] to allow better standardization of our data collection and comparison between our two study sites.
We also collected additional clinical data with standardized definitions to make some adaptations to the WHO-MCS-A classifications to calculate additional estimates reflecting the Sub-Saharan Africa healthcare context. The WHO-MCS-A classification with these adaptations is called the “Sub-Saharan Africa (SSA) adapted WHO-MCS-A classification” for the rest of the paper (cf. Additional file 3).

All data collection tools were pre-tested and procedures tested during a pilot period of one week. Based on experiences recorded during the pre-tests and pilot phase, the study team finalized data collection tools, manuals of procedures and SOP.

The full field study team have been trained for two weeks on the study protocol, Case Report Form (CRF) including detailed definitions and standardized data collection of primary endpoints, databases, SOPs, and manuals. All study staff were also trained on general ethical principles for research and specific ethical procedures for the study including informed consent opt-out processes, confidentiality processes and special training on the topic of abortion. All training included theoretical and practical training phases. Refresher training was conducted by study coordinators according to site needs during the data collection and entry.

In order to prepare our study team to be able to discuss abortion more fully in these legally restrictive and challenging contexts, all study team members participated in a workshop to help them examine their values and attitudes about abortion conducted by trained facilitators(4). These workshops were subsequently rolled out to gynecology ward staff by the study personnel to accompany the introduction of the study, encourage discussion and ultimately reduce barriers to the introduction of the research.

*Registers/medical records screening and inclusion process:*

To identify all potentially eligible patients, trained study clinicians screened daily the registers of each ward where patients with abortion-related complications can present themselves. To do so, they looked systematically at a list of standard key words, including potential symptoms and diagnoses written in the registers. This list of key words was adapted to accommodate the medical vocabulary and acronyms used in each study site. In addition, study personnel participated in the daily clinical staff morning meetings to ensure that all women potentially eligible for the study were identified. They registered the identified potential eligible patients on a paper-based tracking sheet and then looked for the corresponding medical record. Once found, they checked the eligibility of the patient according to inclusion and exclusion criteria fulfilling a standardized eligibility form. If the patient met the

eligibility criteria (including deaths) and there was no opt-out document in the medical record, she was included in the medical record review component. Further information is provided below in "Ethical considerations".

The aggregated number of patients potentially eligible for the study (according to the diagnoses recorded in the hospital registers) but for whom no medical record was opened, were recorded in the standardized "secondary denominators" data collection tool. These women didn't have a medical record because they were treated as outpatients and the existing process in the hospital were to not open a medical record for these patients.

*Prospective medical record review:*

With the help of the health care provider of the women, the trained study clinicians, extracted daily the data from the standardized MSF post-abortion care medical records of eligible women into a standardized case report form (CRF) designed for the study. These CRF were adapted from WHO-MCS-A study forms(5). The study clinician systematically asked the health care provider for each woman if she reported having induced her abortion and/or if they (the providers) found some signs of induced abortion during medical examinations. All unclear and missing information in the medical records was clarified with the woman's health care provider to ensure maximum accuracy and completeness of data in a timely manner.

*Health Management Information System (HMIS) aggregated data:*

The study coordinator also collected aggregated monitoring data necessary for calculating the denominators of some of our secondary study endpoints, including weekly total numbers of deliveries, live births, and admissions in gynecology/obstetrics wards (pregnancy-related admissions) in another standardized data collection form. These aggregated data came from the standardized MSF Maternal HMIS. It includes a standardized post-abortion care medical record, with the same variables definitions and monitoring system (DHIS2).

*Data monitoring and management:*

To ensure a high quality of the data collected, especially on the primary endpoint (severity classification including types of complications), several quality control procedures were conducted.

Every day, the study coordinator (or their deputy) checked all registers against the completed tracking sheet to ensure that no potentially eligible women were missed.

A triple monitoring and validation of the data collection of the CRF was implemented:

- The eligibility form and CRF went through two rounds of on-site monitoring.
    - The first check was done by another study clinician than the one who collected the data or by the deputy study coordinator. S/he checked the completeness of each CRF as well as the accuracy of all collected data against the source document (medical record).
    - The second check was done by the study coordinator. S/he also checked the completeness of the CRFs as well as the accuracy of specific indicators such as the consistency of study identification numbers in all forms completed for one patient in all documents, the eligibility of the woman to participate (including the opt-out status), if all detailed clinical data were collected with accuracy to classify severity (and types of complications) correctly, self or provider report of induced abortion, and coherence of values collected several times in the CRF (systolic blood pressure, hemoglobin, platelet, fluids and blood transfusion given). They also monitored data for outlier values, nonsensical dates and completeness of all treatments given.

- Additional off-site monitoring was conducted for the first 80 patients included in each study site (until agreement rate was >= 98% for all clinical data needed to classify severity) and then, for all near-miss cases and deaths (SMO) as well as 10% of randomly selected other cases. This off-site monitoring was done by the medical doctors of the international committee of the AMoCo study. Scanned copies of the CRF forms and de-identified copies of the patient medical record were shared with offsite monitors via a secure online communication channel (MSF SharePoint only accessible to the study team with secured passwords). This monitoring process involved checking and validating the accuracy of data recorded in the scanned CRF compared with the medical records. Particular attention was paid to the woman's eligibility for inclusion in the medical record review as well as the data collected for the severity classification.

Data were independently double entered using Redcap software at the study site with verification of data entry by regular data checks and a final check of 5% of observations against CRF and questionnaires.

*Data analysis - Definitions and classifications:*

**Maternal death:** Death of a woman while pregnant or within 42 days of termination of pregnancy, irrespective of the duration and the site of the pregnancy, from any cause related to or aggravated by the pregnancy or its management, but not from accidental or incidental causes (ICD-10) (6). The death status is assessed at hospital discharge.

**Abortion-related maternal death:** Maternal death due to spontaneous or induced abortion.

**Maternal near miss:** A woman who nearly died, but survived a complication that occurred during pregnancy, childbirth or within 42 days of termination of pregnancy. Cases are identified according to 25 WHO near-miss criteria. (6)

**Abortion-related near-miss:** A maternal near-miss case that occurs due to spontaneous or induced abortion.

**Live birth:** Birth of an offspring who breathes or shows evidence of life

**Facility-based abortion-related near-miss ratio:** Number of abortion-related near-miss cases that occur in health facility(ies), per 100,000 live births in the same facility(ies). This indicator gives an estimate of the amount of care and resources that would be needed in an area or facility. (6)

**Facility-based abortion-related mortality ratio:** Number of abortion-related maternal deaths that occur in health facility(ies), per 100,000 live births in the same facility(ies). (6)

**Facility-based abortion related mortality index:** the number of deaths that occur in health facility(ies) for 100 severe maternal outcomes (near-miss cases + deaths) in the same facility(ies). It is considered as a quality-of-care proxy indicator in maternal health. The lower it is, the better the quality of care. (6)

*Ethical considerations:*

Ethical approval: The study was approved by the MSF Ethics Review Board (ERB) and the Guttmacher Institute International Review Board, the Central African ethical committee ("Comité scientifique chargé de la validation des protocoles d'étude et des résultats de recherche en santé"), Jigawa State Health Research Committee. The study was conducted in accordance with the Helsinki Declaration and the International Ethical Guidelines for Biomedical Research Involving Human Subjects edited by the Council for International Organizations of Medical Sciences (CIOMS).

Informed consent opt-out process: For the prospective medical record review, a waiver of written informed consent was granted by the 4 ethical committees because, as recommended by CIOMS: 1) the data collected are routine clinical data without identifying information; 2) the research is not possible to carry out with a written informed consent process. In our case, post abortion care is emergency care, and a written informed consent process would have disorganized the emergency care. It would have jeopardized the inclusion of a significant number of women presenting for a short time leading to a risk of low response rate of women with mild complications and a selection bias; 3) the research has an important social value as this was the first study collecting data on magnitude and severity of abortion complications in fragile and conflict settings. Instead, the 4 ethical committees approved the application of an informed consent opt-out procedure. Posters and information notices about the study were available in areas visible to the women presenting for post-abortion care. Pictograms were included in the posters and information notices to help women with low literacy level understanding the information. Health educators provided information about the study in waiting areas and gynecology-obstetric wards where potential eligible women to the study were present. Clinicians in charge of PAC were trained about the study, informed consent opt-out process, and confidentiality principles to verbally inform women about the study and the opt-out option during the clinical visit. If the woman wanted to opt-out, the clinician completed an opt-out form that was inserted in the medical record. A medical record with an opt-out form was excluded from the study. The informed consent opt-out process for women who died was done with the women's family.

Confidentiality: The study data doesn't present results that permit identification of the respondents. Source data was only accessible by designated clinical and research key personnel. Data extracted were de-identified in the data collection tools. The CRF included only the following identifying information: the study individual identification number, the date of the questionnaire completion, the age of the participant without birth date, the name of their neighborhood without address. The list connecting the participant study identification number and his/er personal information (name) was kept only on a paper form in a locked cabinet in a locked study room and was accessible only by the authorized study staff. All study documents were kept confidential by the study team and were stored in a locked cabinet in the locked study room of each site, when not being used by authorized staff. Each member of the study team signed a certificate of confidentiality (CoC). This CoC commits them to following the confidentiality principles and intends to prevent them from disclosing data that can identify a research participant in legal proceedings. The database was stored on password-secured computers in an encrypted folder as well as on a secured Epicentre Sharepoint, only accessible to authorized study personnel. All consent files, the logbook, and filled questionnaires have been repatriated in sealed envelopes to Epicentre Headquarters in a locked cabinet in a locked room because of the volatile context that could not ensure long-term archiving with optimal confidentiality and security. They will be archived for ten years after the database has been frozen and will then be destroyed.

**References:**

1. Hulley SB, Cummings SR, Browner WS, Grady D NT. Designing clinical research : an epidemiologic approach. 4th ed. Lippincott. Vol. Appendix E. Philadelphia, PA 19106; 2013. 81 p.

2. Adler AJ, Filippi V, Thomas SL, Ronsmans C. Quantifying the global burden of morbidity due to unsafe abortion: Magnitude in hospital-based studies and methodological issues. Int J Gynecol Obstet. 2012;118(SUPPL. 2).

3. Calvert C, Owolabi OO, Yeung F, Pittrof R, Ganatra B, Tunçalp Ö, et al. The magnitude and severity of abortion-related morbidity in settings with limited access to abortion services: a systematic review and meta-regression. BMJ Glob Heal. 2018 Nov 1;3(e000692):1–12.

4. Turner KL, Pearson E, George A, Andersen KL. Values clarification workshops to improve abortion knowledge, attitudes and intentions: A pre-post assessment in 12 countries. Reprod Health [Internet]. 2018 Mar 5 [cited 2021 Apr 2];15(1). Available from: https://reproductive-health-journal.biomedcentral.com/articles/10.1186/s12978-018-0480-0

5. Kim CR, Tunçalp Ö, Ganatra B, Gülmezoglu AM. WHO Multi-Country Survey on Abortion-related Morbidity and Mortality in Health Facilities: study protocol. BMJ Glob Heal [Internet]. 2016;1(3):e000113. Available from: http://gh.bmj.com/lookup/doi/10.1136/bmjgh-2016-000113

6. World Health Organization. The WHO near-miss approach for maternal health [Internet]. WHO. Geneva; 2011. Available from: www.who.int/reproductivehealth%0Ahttp://apps.who.int/iris/bitstream/10665/44692/1/9789241502221_eng.pdf

7. Council for International Organizations of Medical Sciences (CIOMS). International Ethical Guidelines for Health-related Research Involving Humans. Biomedical Research. 2016.
