# Supplementary material for: High severity of abortion complications in fragile and conflict-affected settings: a cross-sectional study in two referral hospitals in sub-Saharan Africa (AMoCo study)
Source: BMC Pregnancy Childbirth. 2023 Mar 4;23:143. doi: 10.1186/s12884-023-05427-6 (PMC9985077; doi:10.1186/s12884-023-05427-6)
Supplement: Supplementary file 3 — Additional file 3. WHO-MCS-A and adapted WHO-MCS-A severity classification. [file 12884_2023_5427_MOESM3_ESM.pdf]

Additional file 3: WHO-MCS-A and adapted WHO-MCS-A severity classification

Table additional file 3: Criteria for hierarchal severity classification of abortion-related complications according to WHO-MCS-A with adaptations done in AMoCo (adapted from Qureshi et al. BMJ GH 2021(1))

	Severe Maternal Outcomes				
	**Death**	**WHO Near-Miss Cases***	**Potentially-life threatening complications**	**Moderate**	**Mild**
	**Death**	**Organ dysfunction**	**Conditions**	**Conditions**	**Signs and symptoms on initial assessment**
**WHO-MCS -A Classification**		Cardiovasc	Severe haemorrhage<br>*Blood loss greater than 1000mL, and/or Any bleeding with hypotension, and/or Any bleeding requiring blood transfusion*	Severe bleeding<br>*At least one of the 3 items: 1) Heavy bright red vaginal bleeding (with or without clots), 2) Blood-soaked pads/towels/clothing,3) pallor*	Vaginal Bleeding (including product of conception)
		Respiratory	Severe systemic infection<br>*- Presence of fever (body temperature>38 degrees Celsius) and*<br>*- Confirmed or suspected infection (eg. septic abortion, endometritis, chorioamniotis, generalized peritonitis) and*<br>*- at least one of the following signs:*<br>*1. new/worsened altered mentation,*<br>*2. respiratory rate ≥ 22,*<br>*3. systolic blood pressure ≤ 100 mm Hg*	Abdominal syndrome.<br>*At least one of the 4 items: 1) Abdominal pain/cramping and nausea, vomiting, 2) distended/tense/hard abdomen 3) Shoulder pain 4) Decreased bowel sounds, rebound, tenderness*	Cervix open
		Renal	Uterine perforation<br>*Rupture of uterus confirmed by laparotomy*	Uterine Infection<br>*At least one of the 2 items: 1) Chills, fevers, sweats, 2) Foul smelling vagina discharge +/- History of interference with pregnancy*	Abnormal vital signs<br>*based on temperature, heart rate, systolic/diastolic blood pressure, and respiratory rate*
		Hematologic/ Coagulation<br>*>=5 blood units transfused*			Uterine tenderness
		Neurologic			Abnormal mental status<br>*Agitated, lethargic, comatose*
		Hepatic			Abnormal abdominal examination<br>*Abdominal pain/cramping, nausea, vomiting, Rebounding/guarding. Distended, decreased bowel sounds, tense/hard, tenderness on palpation*
		Uterine			Abnormal appearance<br>*Sick-looking, pallor, jaundice, clammy*
					Cervical Motion Tenderness
					Foul smelling vaginal discharge
					Evidence of foreign body
					Adnexal mass

	Severe Maternal Outcomes				
	Death	WHO Near-Miss Cases*	Potentially-life threatening complications	Moderate	Mild
	**Death**	**Organ dysfunction**	**Conditions**	**Conditions**	**Signs and symptoms on initial assessment**
**AMOCO adaptations for Sub-Saharan Africa**		Hematologic/ Coagulation:<br>*≥2 blood units transfused (instead of ≥5 units)*	Severe haemorrhage:<br>*Same definition with*<br>- *hypotension = SBP <100 mm Hg*<br>- *Adding bleeding + Hb < 4 g/dL*		
			Adding Generalized peritonitis<br>*- T°C>38.5°C and*<br>*- Abdominal guarding (contracture = hard abdomen like roc) or rebound +/- ileus (decreased/no bowels sound, tenderness)*		
			Adding Other intra-abdominal perforations<br>*Evidence of bladder, rectum, bowels mechanical perforation confirmed by laparotomy or examinations*		

* Detailed criteria are in the WHO near-miss approach guidelines(2)
WHO-MCS-A: WHO Multi-Country Study on Abortion (1)

References:

1. Qureshi Z, Mehrtash H, Kouanda S, Griffin S, Filippi V, Govule P, et al. Understanding abortion-related complications in health facilities: results from WHO multicountry survey on abortion (MCS-A) across 11 sub-Saharan African countries. BMJ Glob Heal [Internet]. 2021 Jan 29 [cited 2021 Feb 1];6(1):e003702. Available from: https://gh.bmj.com/lookup/doi/10.1136/bmjgh-2020-003702

2. World Health Organization. The WHO near-miss approach for maternal health [Internet]. WHO. Geneva; 2011. Available from: www.who.int/reproductivehealth%0Ahttp://apps.who.int/iris/bitstream/10665/44692/1/9789241502221_eng.pdf
