# Supplementary material for: High severity of abortion complications in fragile and conflict-affected settings: a cross-sectional study in two referral hospitals in sub-Saharan Africa (AMoCo study)
Source: BMC Pregnancy Childbirth. 2023 Mar 4;23:143. doi: 10.1186/s12884-023-05427-6 (PMC9985077; doi:10.1186/s12884-023-05427-6)
Supplement: Supplementary file 4 — Additional file 4. Facility-based near-miss ratio, mortality ratio and mortality index. [file 12884_2023_5427_MOESM4_ESM.pdf]

Additional file 4: Facility-based near-miss ratio, mortality ratio and mortality index

Table additional file 4: Facility-based near-miss ratio, mortality ratio and mortality index according to SSA adapted WHO near-miss criteria, original WHO near-miss criteria (sensitivity analysis) in both AMoCo study hospitals compared to the WHO-MCS-A in Africa(1) and the Adanikin et al. Nigerian study(2)

	AMoCo Nigeria (AMoCo SSA adapted WHO near-miss criteria)	AMoCo Nigeria (Original WHO near-miss criteria)	AMoCo CAR (AMoCo SSA adapted WHO near-miss criteria)	AMoCo CAR (Original WHO near-miss criteria)	WHO-MCS-A Study (210 facilities in 11 Stable African countries)(1) (Original WHO near-miss criteria)	Nigeria 2019 (42 tertiary hospitals)(2) (WHO criteria adapted*)
Total number of abortion-related near-miss cases	102	23	32	19	264	126
Total number of abortion-related deaths	1	1	2	2	59	65
Total SMO (Severe Maternal Outcome)	103	24	34	21	323	191
Total number of facility live births	6903	6903	2018	2018	NA	91724
Facility-based abortion-related mortality ratio (/ 100 000 live births [95%CI])	14.5 [0.4-80.7]		99.1 [12.0-357.6]		NA	70.9 [54.7-90.3]
Facility-based abortion-related near-miss ratio (/ 100 000 live births [95%CI])	1478 [1206-1791]	333 [211-500]	1586 [1087-2231]	942 [568-1466]	NA	137 [114-164]
Facility-based abortion-related mortality index (Nb of Deaths / 100 SMO [95%CI])	1.0% [0.0-5.3]	4.2% [0.1-21.1]	5.9% [0.7-19.7]	9.5% [1.2-30.4]	18.3% [14.2-22.9]	34.0% [27.3-41.2]

* In Adanikin et al(2). the definition of near-miss case is wider than the original WHO criteria but different from the AMoCo study: they added "severe hemorrhage with severe anaemia <6g/dl" "infection with hyper/hypothermia" and "admission to ICU" as additional criteria for near-miss case => increase of the number of near-miss cases => these extended criteria would overestimate the near-miss ratio and underestimate the mortality index compared to the estimates that would be calculated using the original WHO-MCS-A criteria.

References:

1. Qureshi Z, Mehrtash H, Kouanda S, Griffin S, Filippi V, Govule P, et al. Understanding abortion-related complications in health facilities: results from WHO multicountry survey on abortion (MCS-A) across 11 sub-Saharan African countries. BMJ Glob Heal [Internet]. 2021 Jan 29 [cited 2021 Feb 1];6(1):e003702. Available from: https://gh.bmj.com/lookup/doi/10.1136/bmjgh-2020-003702

2. Adanikin AI, Umeora OUJ, Nzeribe E, Agbata AT, Ezeama C, Ezugwu FO, et al. Maternal near-miss and death associated with abortive pregnancy outcome: a secondary analysis of the Nigeria Near-miss and Maternal Death Survey. BJOG An Int J Obstet Gynaecol [Internet]. 2019 Jun 3 [cited 2019 May 28];126(S3):33–40. Available from: https://onlinelibrary.wiley.com/doi/abs/10.1111/1471-0528.15699
