# Supplementary material for: High severity of abortion complications in fragile and conflict-affected settings: a cross-sectional study in two referral hospitals in sub-Saharan Africa (AMoCo study)
Source: BMC Pregnancy Childbirth. 2023 Mar 4;23:143. doi: 10.1186/s12884-023-05427-6 (PMC9985077; doi:10.1186/s12884-023-05427-6)
Supplement: Supplementary file 5 — Additional file 5. Sensitivity analyses applying the original WHO-MCS-A severity classification. [file 12884_2023_5427_MOESM5_ESM.pdf]

Additional file 5: Sensitivity analyses applying the original WHO-MCS-A severity classification(1,2)**.**

Figure additional file 5: Sensitivity analysis - proportion of women by severity categories using original WHO-MCS-A classification*

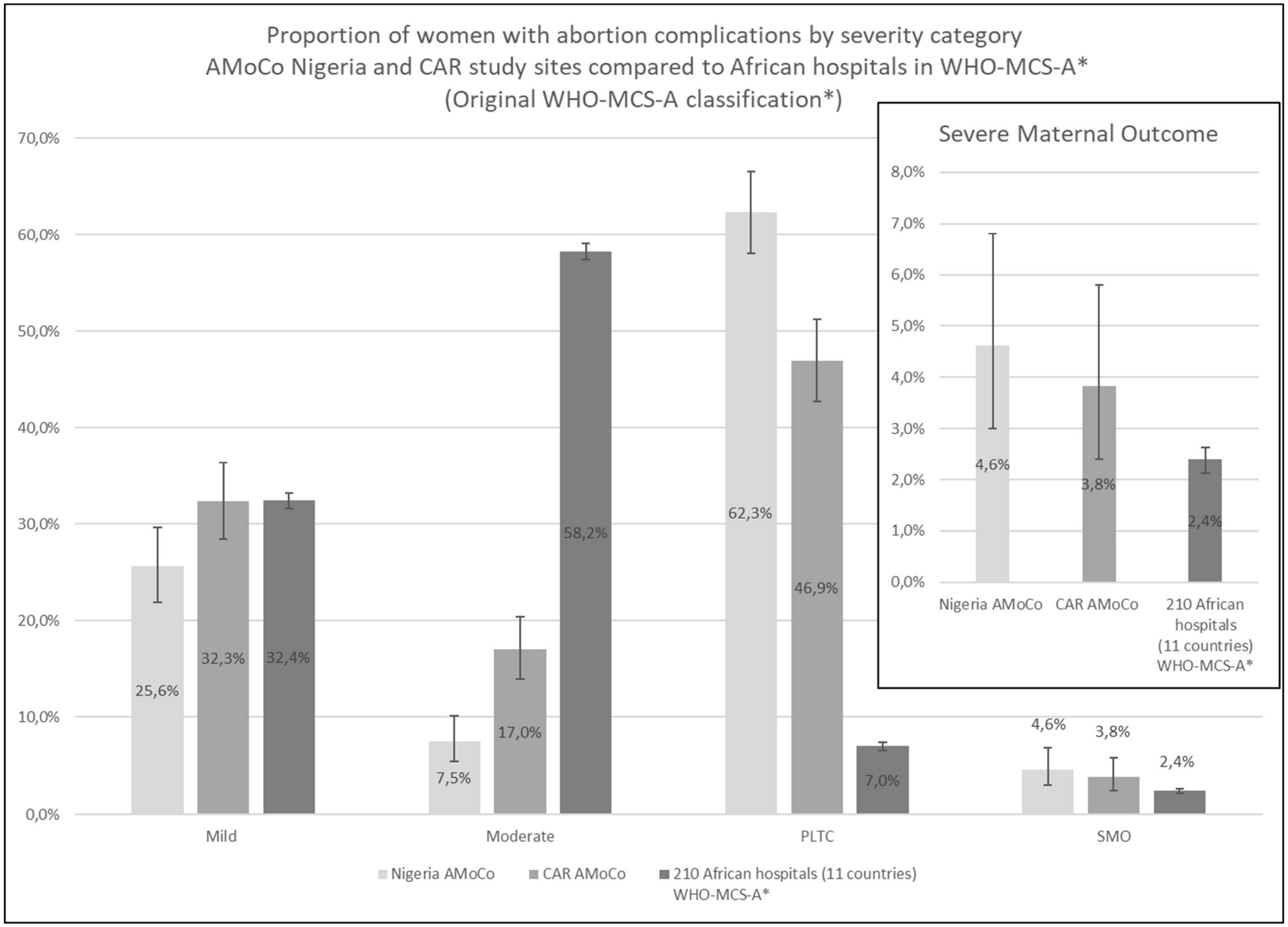

* WHO-MCS-A: World Health Organization Multi-Country Study on Abortion(2)

Table additional file 5: Proportion of women with abortion complications by severity category in AMoCo Nigeria and CAR study hospitals compared to African hospitals in WHO-MCS-A* using original WHO-MCS-A* classification

	Original WHO-MCS-A* criteria								
	Nigeria AMoCo (N=520)			CAR AMoCo (N=548)			210 African hospitals (11 countries) WHO-MCS-A* (N=13657)		
	Proportion	95%CI		Proportion	95%CI		Proportion	95%CI	
**Mild**	**25.6%**	21.9%	29.6%	**32.3%**	28.4%	36.4%	**32.4%**	31.6%	33.2%
**Moderate**	**7.5%**	5.4%	10.1%	**17.0%**	13.9%	20.4%	**58.2%**	57.4%	59.0%
**PLTC**	**62.3%**	58.0%	66.5%	**46.9%**	42.7%	51.2%	**7.0%**	6.6%	7.4%
SMO	**4.6%**	3.0%	6.8%	**3.8%**	2.4%	5.8%	***2.4%***	2.1%	2.6%
Severe complications (PLTC + SMO)	***66.9%***	*62.7%*	*71.0%*	*50.7%*	*46.5%*	*55.0%*	***9.4%***	*8.9%*	*9.9%*

* WHO-MCS-A: World Health Organization Multi-Country Study on Abortion using original WHO near-miss criteria(2)
PLTC: Potentially Life-Threatening Complications
SMO: Severe Maternal Outcome (= near-miss cases + deaths)

References:

1. Kim CR, Tunçalp Ö, Ganatra B, Gülmezoglu AM. WHO Multi-Country Survey on Abortion-related Morbidity and Mortality in Health Facilities: study protocol. BMJ Glob Heal [Internet]. 2016;1(3):e000113. Available from: http://gh.bmj.com/lookup/doi/10.1136/bmjgh-2016-000113
2. Qureshi Z, Mehrtash H, Kouanda S, Griffin S, Filippi V, Govule P, et al. Understanding abortion-related complications in health facilities: results from WHO multicountry survey on abortion (MCS-A) across 11 sub-Saharan African countries. BMJ Glob Heal [Internet]. 2021 Jan 29 [cited 2021 Feb 1];6(1):e003702. Available from: https://gh.bmj.com/lookup/doi/10.1136/bmjgh-2020-003702
